# Impact of Affective Multimedia Content on the Electroencephalogram and Facial Expressions

**DOI:** 10.1038/s41598-019-52891-2

**Published:** 2019-11-08

**Authors:** Siddharth Siddharth, Tzyy-Ping Jung, Terrence J. Sejnowski

**Affiliations:** 1 Electrical and Computer Engineering Department, University of California San Diego, La Jolla, 92093 USA; 2 Institute for Neural Computation, University of California San Diego, La Jolla, 92093 USA; 3 Computational Neurobiology Laboratory, Salk Institute for Biological Studies, La Jolla, 92037 USA

**Keywords:** Cognitive neuroscience, Emotion, Sensory processing, Cognitive neuroscience, Emotion

## Abstract

Most of the research in the field of affective computing has focused on detecting and classifying human emotions through electroencephalogram (EEG) or facial expressions. Designing multimedia content to evoke certain emotions has been largely motivated by manual rating provided by users. Here we present insights from the correlation of affective features between three modalities namely, affective multimedia content, EEG, and facial expressions. Interestingly, low-level Audio-visual features such as contrast and homogeneity of the video and tone of the audio in the movie clips are most correlated with changes in facial expressions and EEG. We also detect the regions associated with the human face and the brain (in addition to the EEG frequency bands) that are most representative of affective responses. The computational modeling between the three modalities showed a high correlation between features from these regions and user-reported affective labels. Finally, the correlation between different layers of convolutional neural networks with EEG and Face images as input provides insights into human affection. Together, these findings will assist in (1) designing more effective multimedia contents to engage or influence the viewers, (2) understanding the brain/body bio-markers of affection, and (3) developing newer brain-computer interfaces as well as facial-expression-based algorithms to read emotional responses of the viewers.

## Introduction

The design of multimedia content such as movies is driven by the director’s assessment of the situation aimed at evoking a particular emotion. For example, action sequences evoking surprise tend to have frequent cuts between the scenes to keep the audience engaged while horror movies evoking fear are usually shot in dimly lighted surroundings to associate the circumstances with negativity. Such cues implicitly affect the audience’s response to the content.

Overwhelmingly, past research aimed at emotion detection/classification has utilized electroencephalogram (EEG) and/or facial expressions^1–3^. In other cases, when such Audio-visual affective cues from the director’s perspective are used in addition to audience’s facial expressions and/or EEG, the goal has been to boost the accuracy of emotion detection/classification framework^4^. Indeed, as a recent detailed survey about recognizing emotions using EEG demonstrates^5^, even after the introduction of multi-modal affective datasets^1,2,6^, the research has not translated from an emotion detection/classification problem to assess the correlation between the Audio-visual cues from the multimedia content and audience’s associated response through changes in the EEG.

When it comes to understanding emotion elicitation and not just detection/classification, there are two parallel trends. First, past research has aimed at designing the optimal feature extraction techniques for mapping the audience’s emotions to either audience’s physiology or to facial expressions. For example, it has been shown that emotion elicitation can be assessed using frontal EEG asymmetry^7^. For facial expressions, there is a vast literature in assessing emotions using the facial action coding system (FACS)^8,9^ that weights the changes in different parts of the face. FACS is based on detecting the changes in facial action units (AUs) such as opening lips, widening eyes, raising eyebrows, etc., associating a score with each AU, and predicting the emotion using the sum of such scores by comparing to a predefined dictionary of possible scores. Such methods do not in any way account for the stimulus that the user is watching, we go a step further to associate AUs and other such features with the emotion-invoking Audio-visual cues present in the stimulus.

Second, researchers have studied emotion elicitation using films by recording and assessing the subjective feedback provided by the audience^10,11^. However, the audience reports evoked emotions as per their perception of the multimedia stimulus. Their physiological recordings are not used to evaluate their responses.

It is between these two trends that this research fits itself. We assess the Audio-visual cues that evoke the emotions in the audience and correlate them with their physiology. We do this by extracting feature cues from the multimedia content such as shot duration, visual excitement, color energy, audio tone, etc. that are known to evoke an emotional response in the audience. We then use canonical correlation analysis (CCA)^12^ between such cues and features from audience’s (neuro)physiology. In this way, we generate a model to represent the features from both worlds in a single domain.

Subsequently, we perform the analysis on the model to provide insights into the regions from the face and brain (i.e. the audience’s physiology) that are most correlated with emotions. We also perform such analysis to detect what type of Audio-visual cues are most effective in evoking changes in the human physiology.

Finally, we correlate the components of the joint model generated from audience’s physiology and Audio-visual cues with audience’s recorded emotional response. We believe that the insights we obtain from this study will help in designing more effective multimedia contents to engage or influence the viewers, understanding the brain/body bio-markers of affection, and developing newer brain-computer interfaces as well as facial expression-based emotion classification algorithms to read emotional responses of the viewers.

## Results

### Relationship between Audio-visual Cues and EEG Cues

Canonical correlation analysis (CCA)^12^ is a method for exploring the relationship between two multivariate sets of vectors. This method can be used to compute which variables and their combinations in a set are most correlated with those in another set. Since all our feature cues are correlated with human emotions, it allows us to perceive which of those cues contribute the most towards emotion elicitation and classification. We first used CCA to find the relationship between changes in the EEG cues (theta, alpha, and beta EEG band power-spectrum density and conditional entropy across brain regions) and over 34,000 instances of features from the Audio-visual cues representing human emotion. These EEG and Audio-visual cues are detailed in the Methods section below and they capture the low- and high-level information from the multimedia content. Specifically, low-level cues are those which are not directly perceived consciously by humans such as texture of a video content and pitch of the sound while high-level cues are the ones which are associated with human cognitive capabilities such as speech recognition and how visually exciting a scene is based on the temporal speed of the content. The method was applied to the data from each subject separately and the aim was to find the leading Audio-visual cues that modulate the EEG spectra. This would help in pinpointing the kinds of stimulus that strongly affects human emotions.

**Figure 1:**
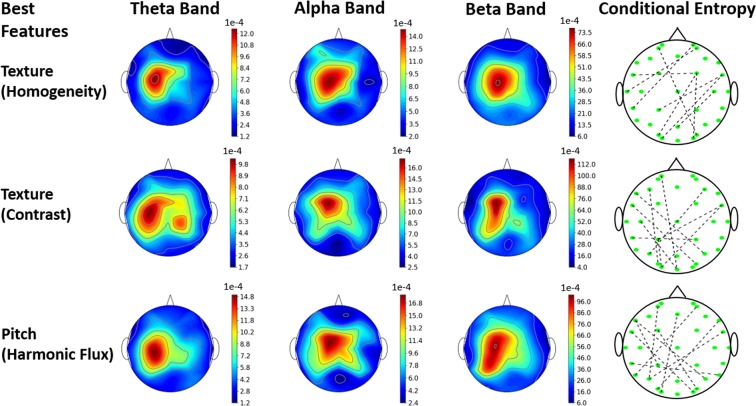
The three leading Audio-visual Cues, Texture-based Homogeneity, Texture-based Contrast, and Pitch-based Harmonic Flux, associated EEG spectral changes in the theta, alpha, and beta bands, and top 10 Conditional Entropy EEG channel pairs.

Figure 1 shows the corresponding EEG coefficients for each of the three frequency bands for the first three most correlated Audio-visual cues. It is notable that among the 30 Audio-visual cues, the three most correlated cues are low-level ones i.e. based on video texture and audio pitch rather than high-level ones such as the number of shots and voice probability. These texture- and pitch-based features usually model the background imagery and sound in multimedia contents. We also show the corresponding brain regions connected by 10 most correlated conditional entropy cues. It is also notable that the most active areas in the brain are present across the C3, Cz, and C4 electrodes i.e. in the central section of the 2D brain representation. Finally, looking at the values on the three color-bars, we see that beta-band was affected by the Audio-visual cues much higher than the theta and alpha bands. The p-values calculated by the paired-sample t-test for Texture (Homogeneity) for theta-alpha, alpha-beta, and theta-beta pairs at the Cz electrode (located at the center of the 2D brain image) were 0.16, 0.002, and 0.005 respectively, for Texture (Contrast) were 0.178, 0.00008, and 0.0001 respectively, and for Pitch (Harmonic Flux) were 0.16, 0.00015, and 0.00018 respectively. These statistical tests showed the significance of beta power being statistically different than theta and alpha ones whereas the Fig. 1 shows the beta power to be significantly larger than the theta and alpha power. The results suggest that the beta-band power in EEG recordings might be used for distinguishing affective states to a significantly more extent than the theta and alpha EEG band power.

### Relationship between Audio-visual Cues and Facial Expression Cues

**Figure 2:**
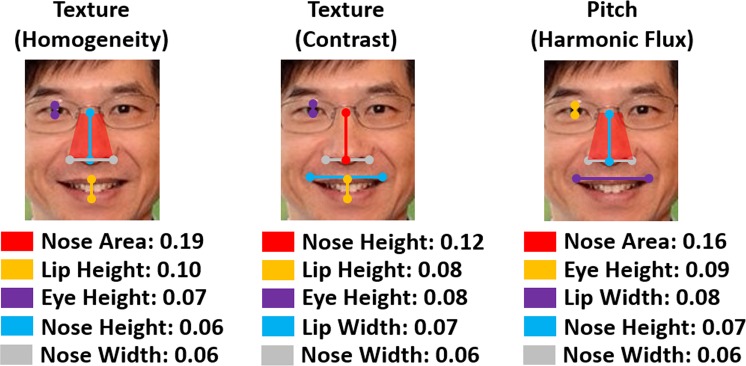
The same three Audio-visual Cues as with the EEG (Texture-based Homogeneity, Texture-based Contrast, and Pitch-based Harmonic Flux) were found to be most correlated with Facial Expressions. We plot above the five best associated Facial Expressions Cues and denote weights of their associated CCA coefficients.

Similar to the EEG cues above, we performed CCA between Audio-visual cues and Facial Expression Cues. By this process, we wanted to find which cues from each of these modalities are most correlated with each other. Similar to the EEG, we found that among the 30 Audio-visual Cues, texture-based and pitch-based low-level cues affect facial expressions most. Figure 2 shows the five most correlated Facial Expression cues corresponding to each of the leading Audio-visual Cues. In all three cases, the nasal region is the most representative part of the human face. This is because either the area of the nose or the height of the nose which modulates in a 2D camera image as per vertical bending of the human face contributes significantly more than other facial regions. After the nasal region, lips and eye-opening contribute most towards the Audio-visual Cues.

### Relationship between EEG Cues and Facial Expressions Cues

**Figure 3:**
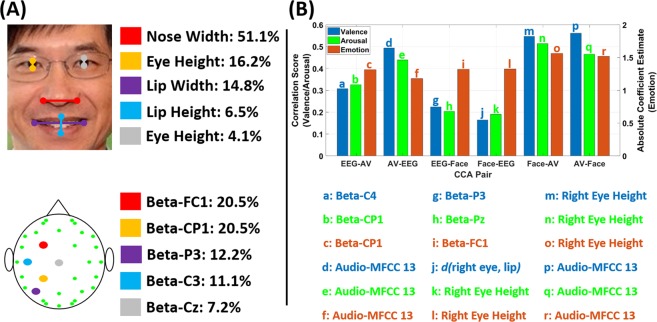
(**A**) (Top) Five most contributing Facial Expressions Cues (note that the width of nose alone contributes towards half of the total) and (Bottom) Five most contributing EEG Cues (note that all contributions are by EEG Beta-band Power only) and (**B**) (Top) Correlation score between each CCA pair (EEG-AV denotes the correlation between EEG projected from the joint EEG-AV space and Valence/Arousal (Left y-axis) or Emotion (Right y-axis)) and (Bottom) the cue with the largest correlation score for each bar graph. *d(a,b)* denotes the Euclidean distance function.

Having found the correlation between the multimedia contents that the users are watching and their physiological responses, we sought to find the relationship between their physiological response (EEG) and behavior (Facial Expressions). To this end, we applied the CCA to the EEG Cues and Facial Expression Cues. Figure 3(A) shows the results of this analysis. As a compilation of the above two results, we again see that the nasal region contributes most towards the Facial Expression Cues, followed by eye height and lip width and height. Similarly, all five most contributing EEG Cues are from the Beta-band EEG power in the left central areas. This is also consistent with previous results which have shown that this brain region represents human valence and arousal very well^3^.

### Correlation between the Audio-visual, EEG, and Facial Expressions Cues with subjective responses

As discussed in the Methods section, for each multimedia clip, the subject reported his/her valence (between 1 to 9), arousal (between 1 to 9), and emotional tag (one of twelve emotions). After calculating the CCA projection between each pair of the three kinds of feature cues (Audio-visual, EEG, and Facial Expressions), we projected back the joint CCA space to the feature cues. For Arousal/Valence, we then calculated the Pearson correlation between the CCA projections and behavior data (user-reported labels) while for Emotions, we fit a linear regression model^13^ between CCA projections and behavior data (user-reported labels). This is done differently for these three affective measures since Valence/Arousal are distributed on a scale of 1 to 9 denoting their intensity whereas Emotions were tagged by the users directly to be one of the many categories. Hence, we fit a linear regression model on users’ reported emotions and plot the highest absolute value of the coefficient estimate. In this way, for Valence/Arousal/Emotion, we are able to plot how closely did our CCA components method correlate with users’ behavior data.

Figure 3(B) shows that the projections along the top CCA components between Facial Expression Cues with Audio-visual (AV) Cues provide the highest correlation with the behavior data (two rightmost sets of bars). Below, we show which cue in each of the three modalities provides the highest correlation with the user-reported affective measures. Each set of bars such as Face-AV and AV-Face denote the projection of either Facial Expressions Cues and Audio-visual Cues from the joint Face-AV Cues space that was used to calculate the correlation with behavior data. For each such correlation, we also list that which of the cues from that modality provided the highest correlation.

Thus, we see that EEG beta-band power near the centro-parietal region (e.g. C4, Cp1, etc.) has the highest correlation with behavior data (bars labeled as a, b, and c), while MFCC feature 13 i.e. the last audio MFCC coefficient provides the highest correlation among the Audio-visual Cues with emotional responses (the bar labeled as d). This MFCC coefficient is related with very fine pitch and tone information since it is the highest coefficient among those generally used in speech processing research. Finally, among the Facial Expression Cues, the height of the right eye i.e. the vertical distance of the eye-opening (bars labeled as k-o) has the highest correlation with user-reported affective measures in most cases. Hence, this part of our analysis bridges the gap between the cues from the three modalities that we analyzed and users’ self-reported affective measures (behavior data).

### Correlation between EEG and Facial Expressions through Deep-Learning Network Cues

All the Cues that we analyzed above were extracted by the methods reported in previous academic research or were designed by hand. But, with the advent of deep-learning research in previous years, it is now possible to let the convolution networks decide which feature cues to extract and use for a particular application. To this end, we utilized a recently developed technique to convert time-domain EEG data into image-based representation^3^. Utilizing this method, we generate a single (RGB) color image representative of EEG power in all the three frequency bands (theta, alpha, and beta) for each of the 15 s multimedia clip. Similarly, we used an image fusion^14^ method to represent the face region in successive frames with a single frame.

While deep-learning research on computer vision data has a wide breadth of research, we utilized the knowledge from two very recent publications detailing the use of deep-learning for EEG^15,16^ to train a VGG-16^17^ network-architecture-based model. This network consists of 16 weight layers with millions of parameters and have shown to work very well for various image classification problems. We randomly used 90% of our total trials for training the network and remaining for for testing the activations. Similarly, we train another VGG-16 model on face images from the affective dataset. Our goal behind training these networks was to extract features varying from low to high level that is not possible for humans to visualize and design.

**Figure 4:**
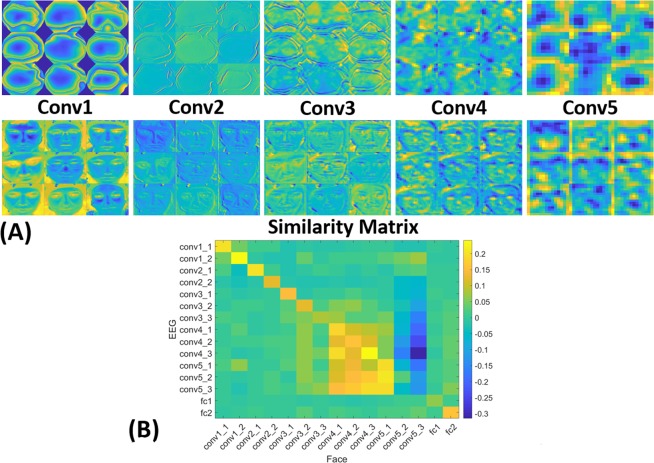
(**A**) A sample feature representation of different layers’ activations in the VGG-16 network for nine example EEG and Face data inputs and (**B**) A similarity Matrix shows the correlation at each layer between EEG and Face representations in the VGG-16 network.

Figure 4(A) shows nine sample inputs to the network and the activations of five successive convolution layers. We can visualize that as the number of layers increases, the activations’ representation inside the network becomes more low-level i.e. higher resolution. Thus, going through all layers of the network, it is possible to capture the full variation in such feature representation. For each such layer for all input images, we generate a representation similarity matrix (Fig. 4(B)) between EEG and Face images similar to what is done for Magnetic Resonance Imaging (MRI) data^18^. We show the correlation between 15 of the 16 layers’ activations of the VGG-16 matrix (since the last layer corresponds to classification and hence was discarded). The highest correlation in the similarity matrix is located in the middle layers, which means that those layers (conv4 and conv5) represent the activations that are highly correlated in the eeg- and face-representation learned by the deep-learning network. It is also notable that because Conv4 and Conv5 layers are just before the fully connected ones, they contain the most discriminative features as one progresses from Conv1 to fc2 layers. This would mean that such low-level discriminative features could be more useful when utilized for affective analysis just like we found that low-level Audio-visual features were more discriminative than high-level ones in influencing users’ EEG and facial expressions.

## Discussion

Past research in affective computing has mostly focused on emotion classification using various Audio-visual or bio-sensing modalities^1,2^. A severe limitation of such research has been its inability to boost classification accuracy to human-like levels. Another limitation of previous research has been its inability to demonstrate which cues related to Facial Expressions or EEG are most correlated with human emotions. This is because FACS-based facial action units^9^ are hand-coded cues that were chosen since they represent specific muscle movements or set of muscle movements and are by definition mapped to particular emotions. Similarly, the most commonly used EEG power spectrum features have been used blindly for emotion classification without any insight into which brain regions or frequency bands actually contain the most affective content. It was to address these limitations that we sought to understand the relationships between multimedia content itself with users’ physiological responses (facial expressions/EEG) to such content.

By the means of Canonical Correlation Analysis (CCA)^12^, we analyzed these three modalities after extracting cues representative of affective information from each of them. We found that for both EEG and facial expressions, the Audio-visual cues that are most correlated with them are those that represent low-level features found in the image background/background music such as texture and pitch. This is a useful insight into the design of such content to intentionally customize the content’s image background and background music for invoking particular affective responses.

The other side of the coin from the above analysis is that most of the spectral features from the EEG are concentrated at/around the Cz electrode position (center of the 2D brain image) and are present in the beta frequency band. This is an insight that can help design the next generation of wearable EEG headsets for affective applications since focusing on these brain regions and frequency band can provide most of the affective information and thus sensors covering the whole brain may not be needed. Similarly, for facial expressions, we found that the nasal region followed by eye-height i.e. how much the user opens his/her eyes, and lips are most representative of changes in affective multimedia contents. Hence, new features can be designed and added to the FACS method to utilize more information from these three facial regions only (for example, the nasal area and nose height representing vertical head tilt in a 2D image are not currently used as facial AUs). We believe that the changes in features across different analyses (e.g. nasal region vs right eye height as the most correlated features) happen because different kinds of affective contents may induce different affective states in human physiology. Thus, it is not possible for a single brain region or facial region to be universally representative of every affective state of the subject^19^.

When we quantified the relationship between facial expressions and EEG, we again found that the nasal region followed by eye-height and lips are most representative of changes in facial expressions while the beta-band power distributed at/near the central region is most representative of changes in the EEG while the participants watch the multimedia contents. Similarly, previous research has shown that beta-band activity reflects emotion and cognitive processes very well^20^. In fact, the top five features in facial expressions and EEG alone constitute more than 90% and 70% weightage respectively compared to all possible 30 facial expression cues and 96 EEG cues. It suggests that utilizing only these five cues from both modalities can provide much of the affective information. Finally, the high EEG-activity at/near the center of the brain was also consistent with previous work that showed these brain regions are also most representative of changes in human valence and arousal^3^.

When we projected back the joint CCA space of these modalities to reconstruct the cues from each and correlated them with users’ subjective valence/arousal/emotion ratings, we found the highest correlation (with values above 0.5 even when valence/arousal ratings have a high resolution since they are divided on 9/9 point scale respectively) for Audio-visual cues followed by that for facial expressions cues. In a way, this is intuitive since the Audio-visual cues extracted from the multimedia content itself should contain most of the affective information. Subsequently, this also proved for us as a way to show that the extracted Audio-visual cues do represent the affective information as perceived by the users. Facial expressions cues are second-most correlated whereas EEG cues are least correlated with users’ responses. This is probably due to the fact that image data does not have as much noise/artifacts as are present in EEG data. We also again find that low-level Audio-visual cues contribute the most towards high correlations with users’ subjective ratings. This shows that our emotions are affected much more by “background” cues in this context such as pitch and texture of the scene rather than “foreground” human speech and how fast the video content changes from frame to frame.

Deep-learning networks utilize many convolution layers to extract features that can be characterized by an increasing level of complexity. We used such a deep convolution network^17^ to extract features of various complexity from EEG and face data. We then generated a similarity matrix to represent the correlations between each pair of convolution layers between the modalities. We found that the highest correlation was present at the fourth and fifth convolution layers. This points out that the information from raw EEG and face data has to be first sufficiently processed to generated these “optimal” low-level features since they are most representative of joint changes in the two modalities while watching affective multimedia contents. We present these results as a starting point to take this research further into actually interpreting such low-level features extracted by the convolution network and then use them for emotion elicitation and classification problems.

To conclude, our findings provide various insights into the affective cues of these three modalities, both for designing affective multimedia content as well as for designing the next generation of EEG- and vision-based emotion classification systems. Through the above-mentioned analysis, we show that low-level Audio-visual cues are most representative of human emotions, even when they may be subconsciously influencing our emotional states. In the future, we would like to take this work forward towards increasing the resolution of affective cues pertaining to emotions such as by associating particular EEG/facial expression cues with happiness, joy, fear, surprise, etc.

## Methods

In this section, we describe the dataset and the analytical tools we used for this study.

### Dataset

We used the publicly-available MAHNOB-HCI Dataset^6^ for this study. The dataset contains multi-modal data i.e. frontal video, EEG, electrocardiogram (ECG). galvanic skin response (GSR), etc. from 27 users watching 20 short movie clips. These movie clips vary between 35 and 117 seconds in duration. Each clip is reported by the subject on a scale of integers from 1 to 9 to denote their arousal and valence according to the emotion circumplex model^21^. Additionally, the users also tag their felt emotion into one of the twelve categories such as anger, disgust, fear, joy, anxiety, etc. The clips have been chosen to evoke a certain emotional response in the audience. For example, clips from the *Mr. Bean* character and funny cat videos have been used to evoke amusement, clips from horror movies such as *Hannibal* and *Silent Hill* have been used to evoke disgust/fear response associated with horror, and clips from weather news coverage have been used to evoke a neural response in the audience.

The choice of this dataset was made because the dataset captures frontal videos at a high resolution of 780 × 580 pixels and EEG with a high-density headset (32 channels) at a high sampling rate (256 Hz). Furthermore, the datas*et al*so provides the 20 movie clips that were used as stimuli for evoking the emotional responses in the audience. Since the dataset contains only about 540 trials, we windowed each fifteen-second section of the data and advanced it by one second. In this way, we augment the dataset to get more than 34,000 trials for each data modality.

### Audio-visual cues

Here, we present the cues that we extracted from each movie clip (stimulus) for the analysis with the audience’s response. We extracted eleven cues from the video and nineteen from the audio.

#### Audio cues

We extracted nineteen audio cues from each multimedia clip. Almost every such multimedia clip has background music playing with selected musical instruments to associate the scene with a particular emotional context. The presence of the human voice in the clip also provides another cue that some high-level information is being conveyed by communicating through the voice.

MFCC Features: Acoustic characteristics in music are associated with the expression of emotions^22^. To assess spectro-temporal characteristics that represent the quality of sound making a particular type of music different from another, we extract thirteen Mel-frequency cepstral coefficients (MFCC) as features. These features characterize the spectral shape of the sound and have been used in previous studies too for analysis with emotions^4^. To obtain these features, we used the publicly available music information retrieval toolbox, MIRToolbox^23^.

Loudness and Loudness Range: Loudness depends on the physical intensity of sound as well as the frequency and duration. Loudness, in general, is associated with emotional arousal as well as the perception of loudness itself can be influenced by the emotion^24^. Thus, we calculated the average loudness for each audio clip as well as the loudness range (LU units).

Voice Probability: The presence of human speech in a clip is a high-level feature depicting that some information is being conveyed through communication in addition to the background sound. Thus, we calculated the probability of having human speech in each audio clip using a commonly-used statistical model^25^.

Pitch Features: To extract features related to the fundamental frequency of sound, we extracted pitch-based features. Pitch can model the harmonic as well as the melodic aspect of the music and is thus a good low-level feature. It has also been shown that the systematic changes in pitch level can affect the experience of pleasantness and arousal^26^. Thus, we extracted three features based on the pitch: keyclarity, mode, and harmonic flux^23^.

#### Visual cues

We extracted eleven cues based on the video from each multimedia stimulus. These features capture low-level information such as texture and color as well as high-level information such as shot duration and visual excitement. These visual cues are generally used while producing movies and have been shown to capture affective information^27^.

Visual Excitement: The amount of motion in a video plays a significant role in our perception of the cinematic experience and affective response, particularly the amount of arousal^28^. Thus, we calculated visual excitement based on the average number of pixels that change between successive frames according to human perception^27^.

Shot Duration Features: As mentioned before, the duration of the shot, also known as the pace of the movie, changes as per the type of content. Rapid changes in shots from multiple camera angles induce a degree of excitement far more effectively than a long duration shot^29^. Thus, we calculated the number of shots and average shot duration for each video clip using the open-source PySceneDetect tool^30^.

Lighting Key Features: In the cinematographic perspective, lighting is an extremely powerful tool since it can be used to affect the audience’s emotions by manipulating the mood of a scene. Dim lighting is generally used to convey negative themes in the content whereas an abundance of illumination generates joy and warm atmosphere around the scene^31^. We first converted the RGB color space frames to LUV color space for separating the illuminance information of each frame. Subsequently, we calculated the lighting key features for each frame to measure the median general level of light and the proportion of shadow area in the clip^32^.

Color Energy: Psychological studies on color have demonstrated a high correlation between valence and arousal with brightness and saturation of the colors respectively^33^. We calculated color energy based on the brightness, saturation, and area occupied by the colors in a frame. This measure is defined as the product of the raw energy and color contrast^27,31^.

Texture-based Features: Visual detail has been shown to affect the audience’s emotional distance to a scene such as by varying the camera distance of the shot^34^. We transformed each video frame to generate the grey level co-occurrence matrix (GLCM) which models the distribution of co-occurring pixel values. This matrix can map the variations in texture such as gray-level contrast across the frame^35^. We then calculated four features on this GLCM matrix and average them across all the frames in a clip: contrast, correlation, energy, and homogeneity. These features map local variations in GLCM, the joint probability of occurrence of the pixel pairs, the sum of squared elements of the GLCM, and closeness of elements distribution from the diagonal of the GLCM respectively. Finally, we also calculated texture-based features based on the saturation of colors by calculating the proportion of pixels whose saturation in the normalized HSV color space exceeds 0.2 as the threshold.

**Figure 5:**
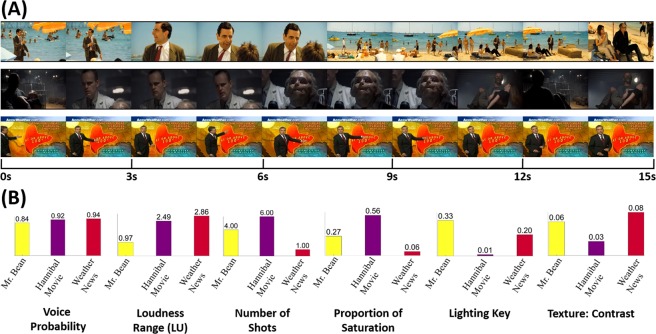
(**A**) 15-seconds examples (showing one frame for every 1.5 seconds) of three multimedia clips: *Mr. Bean* (above), *Hannibal Movie* (center), and *Weather News* (below) and (**B**) Six of the thirty Audio-visual cues are plotted to show the variation of features across the three clips.

In Fig. 5 we show an example of calculating these Audio-visual cues through three 15 s multimedia clips from the dataset. As we would expect, the voice probability is highest for *Weather News* clip but it has been filmed in only one shot whereas lighting key is least (dim lighting) and saturation is the highest for the *Hannibal* movie clip i.e. horror scene.

### EEG Cues

We first cleaned the EEG data of various types of noise/artifacts such as due to eye blinks, muscle movements, electromagnetic disturbances, etc. using the Artifact Subspace Reconstruction (ASR) pipeline^36^. After cleaning the data, we extracted two distinct types of EEG features. The first kind of EEG features is the traditionally used power spectrum density (PSD) for each of the 32 EEG channels. We computed the power spectrum density for three EEG frequency bands namely, theta (4–7 Hz), alpha (7–13 Hz), and beta (13–30 Hz). We chose these three EEG frequency bands because they account most information towards human cognition^37^ and thus have been most commonly used in affective research^5^. We did not use other EEG bands such as the high-frequency gamma band (above 30 Hz range) since the present literature does not support its association with human consciousness (highly associated with affective states)^38^. For each 15 s clip, we averaged the EEG-PSD features across the whole clip. As a result, we obtained 96 EEG-PSD features for each multimedia clip.

The above EEG-PSD features are topographically localized and hence do not account for the variations in EEG across the human brain. Emotion elicitation is a complex process in which more than a single brain region may be involved. Previous research^3,39^ has shown that using features that capture changes in EEG across different brain regions can boost the performance for emotion classification. Thus, for each pair of EEG channels (32 EEG channels forming 496 such pairs), we computed conditional entropy features^39,40^. These features are calculated based on the mutual information content between each sensor pair i.e. how much information is contained in one EEG channel given the information from another EEG channel. In this way, we extracted 496 features for each 15-second multimedia clip to represent changes across different regions of the brain.

**Figure 6:**
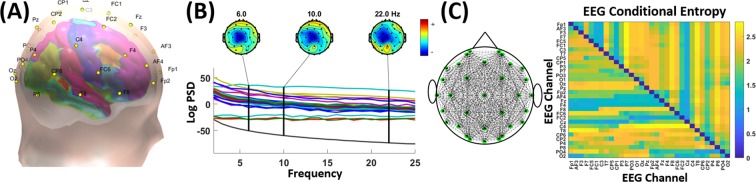
(**A**) EEG channel locations on the scalp used to compute local EEG-PSD Features, (**B**) An example of EEG-PSD heat maps calculated at three frequencies, and (**C**) Conditional Entropy pairs across the brain shown from the 2-D top-view of the scalp with the EEG Conditional Entropy feature matrix.

Figure 6 shows the 32 EEG channel locations that are used to calculate the localized EEG-PSD features. The 3-D head model was generated using the open source headModel toolbox^41^. We also show examples of PSD heat-map at three example frequencies, and the 496 channel pairs used to calculate conditional entropy and the conditional entropy matrix so calculated. To reduce redundancy, we only use the lower half of the matrix values i.e. one-way conditional entropy.

### Facial Expressions Cues

For each face video clip, we first detected the user’s face and extracted the face region from each camera frame using Haar-like features by utilizing the Viola-Jones object detector^42^. While running the face detection algorithm, we excluded the ends of the image and placed a threshold of minimum face size to be 50 × 50 pixels to remove false positives. We then used state-of-the-art automated Chehra algorithm^43^ to extract the face region to obtain 49 facial landmarks. These facial landmarks are located at the most expressive regions on the human face.

**Figure 7:**
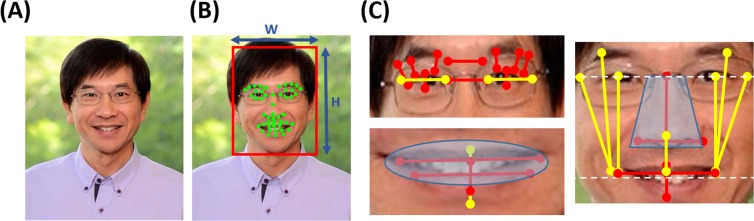
Illustrating the working of our algorithm through (**A**) a sample image frame, (**B**) extracted face region and locating facial landmarks, and (**C**) 30 distance/area features computed based on the facial landmarks. To account for different face sizes and distance from the camera, all features were re-scaled based on face width (W) and face height (H). A sample image was used to generate this figure in order to respect the privacy of the users who participated in the study.

We utilized these 49 facial landmarks to calculate 30 features based on the distance and area calculated using these landmarks such as the vertical distance of the eye-opening, the distance between the eyebrow to the lips, horizontal and vertical distances between the ends of the lips, the area of the mouth and nose, etc. We show these facial landmarks and features in Fig. 7. Most of these features are the same which are used in the calculation of facial action units (AUs)^9^ and thus have been used for emotion recognition through facial expressions. Some other features were selected by hand. Because the shape of the face vary across human beings and the area taken by the facial region in an image vary with distance from the camera, we normalized all such features based on the width and height of the detected face. Since each multimedia clip contains many frames, we first used wavelet decomposition across all face images^14^ to fuse all such face images to one face representation. It is on this image which contains the “representative” facial expression across the multimedia clip that we calculated the above features.
